# Training the Next Industrial Engineers and Managers about Industry 4.0: A Case Study about Challenges and Opportunities in the COVID-19 Era †

**DOI:** 10.3390/s21092905

**Published:** 2021-04-21

**Authors:** Arriel Benis, Sofia Amador Nelke, Michael Winokur

**Affiliations:** Faculty of Industrial Engineering and Technology Management, Holon Institute of Technology, 52 Golomb Street, Holon 5810201, Israel; sofiaa@hit.ac.il (S.A.N.); michaelw@hit.ac.il (M.W.)

**Keywords:** Industry 4.0, industrial engineering, educational activities, education, project-based learning, business intelligence, internet of things, project management, cyber-physical systems, COVID-19, coronavirus, digital health

## Abstract

Training the next generation of industrial engineers and managers is a constant challenge for academia, given the fast changes of industrial technology. The current and predicted development trends in applied technologies affecting industry worldwide as formulated in the Industry 4.0 initiative have clearly emphasized the needs for constantly adapting curricula. The sensible socioeconomic changes generated by the COVID-19 pandemic have induced significant challenges to society in general and industry. Higher education, specifically when dealing with Industry 4.0, must take these new challenges rapidly into account. Modernization of the industrial engineering curriculum combined with its migration to a blended teaching landscape must be updated in real-time with real-world cases. The COVID-19 crisis provides, paradoxically, an opportunity for dealing with the challenges of training industrial engineers to confront a virtual dematerialized work model which has accelerated during and will remain for the foreseeable future after the pandemic. The paper describes the methodology used for adapting, enhancing, and evaluating the learning and teaching experience under the urgent and unexpected challenges to move from face-to-face university courses distant and online teaching. The methodology we describe is built on a process that started before the onset of the pandemic, hence in the paper we start by describing the pre-COVID-19 status in comparison to published initiatives followed by the real time modifications we introduced in the faculty to adapt to the post-COVID-19 teaching/learning era. The focus presented is on Industry 4.0. subjects at the leading edge of the technology changes affecting the industrial engineering and technology management field. The manuscript addresses the flow from system design subjects to implementation areas of the curriculum, including practical examples and the rapid decisions and changes made to encompass the effects of the COVID-19 pandemic on content and teaching methods including feedback received from participants.

## 1. Introduction

Training the next generation of industrial engineers and managers is a constant challenge for academia. Course contents must be up-to-date and cover all current and predicted development trends. In addition, they must always be relevant with the end-to-end product chain, from client requirements to maintenance via design and production. The COVID-19 pandemic has induced a significant number of challenges to industrial activities and their management. Graduates of today’s academic programs will have to handle tomorrow’s challenges from the technological, economic, medical, and industrial perspectives. Therefore, to be relevant, program contents had to be adapted to the new reality in a short time. In this manuscript, we expose the ante and during COVID-19 pandemic upgrade of the curriculum of the Bachelor degree in Industrial Engineering and Management (IEM) at the faculty of Industrial Engineering and Technology Management (IETM) of the Holon Institute of Technology (HIT), Israel. This manuscript focuses on the academic issues related to the Industry 4.0 requirements, which are at the forefront of the needs for constant adaptation [1] in all phases from system design through implementation and activity report.

Moreover, we center our attention on the challenges encountered while delivering technological courses during the COVID-19 pandemic and the need to transfer to a regime of remote-teaching to comply with regulations that were imposed in real time and support the urgent need for social-distancing.

One specificity of the B.Sc. program at HIT we must present up front, since it has a significant impact on our methodology, is the fact that two parallel tracks co-existing during the first three years of the B.Sc., the regular track and the evening track. The first one offers five days a week teaching during the two regular semesters (fall and spring, each one of 13 weeks long). Generally, regular track students are not working. The evening track relies on participants working in parallel with their degree where courses are given two evenings a week and on Friday morning (6 h each time) all over the year (two regular semesters and summer semester). The fourth-year is unified for both regular and evening tracks students learning evenings and weekends.

During the four years of the programs, the students are progressively trained for starting their careers in positions such as industrial engineers, data analysts, and information system managers with an emphasis on the technology management aspects. Moreover, these positions require close interaction and cooperation with other specialists such as electrical and software engineers, designers, trainers, domain-experts (e.g., healthcare professionals, food technologists, defense and security specialists), and the ability to manage multidisciplinary projects. Therefore, the IEM curriculum must be updated continually to provide theoretical knowledge and practical experience. The focus is on ubiquitous technologies such as Industrial Internet of Things (IIoT), Automation, Robotics and Embedded Systems, and Applied Informatics (including Visual Programming, Big Data Analytics, and Business Intelligence) [2,3]. The study program includes courses that strongly emphasize the technologies mentioned above.

More particularly, the nowadays-trained next generation of industrial engineers that will manage Industry 4.0 systems and related-projects will need to understand how these new production environments are impacted and reciprocally influence others [4]. This multidisciplinary understanding must allow prioritizing the business development process [5] by clearly understanding the business opportunities of their company taking into account sustainability challenges [6] and dealing with them to allow continuous improvement of business performance [7].

A “learning by experience” policy has been implemented since 2018 to encourage the IEM students to develop their soft and practical skills. One of the main objectives is to develop the students’ project-based learning capabilities by involving them in real-world-oriented tasks and projects during each course and over time by linking projects in different courses. Moreover, this policy facilitates the interactions between the faculty’s laboratories for operating an integrated cluster of educational and research laboratories [8]. In addition to new courses such as “Introduction to the Internet of Things (IoT)” [9], and as a part of this change, the courses have increasingly been using Project-Based Learning (PBL). The PBL allows dynamically involving students in applied research-oriented and real-world tasks. The PBL aims to drive students’ self-learning to develop more in-depth skills and capabilities [10].

During the first months of 2020, the SARS-CoV-2 virus spread worldwide and induced the COVID-19 pandemic. According to the World Health Organization (WHO), one year later, as of 31 January 2021, there have been 101,561,219 confirmed cases of COVID-19, including 2,196,944 deaths over 220 countries, areas, or territories [11]. This disruptive event had a considerable impact on public health, industry, the economy, and education [12,13,14]. In the specific case disclosed in this manuscript, the students of the regular and evening tracks have been differentially impacted.

In March 2020, to limit the spread of infection, social distancing was enforced [15,16], including restricting the number of attendants at in-person gatherings and advising to keep a distance of 2 m between individuals [16,17]. On 12 March, the overall education system in Israel was closed by the government order and gradually moved to distant online teaching [18]. Up to 21 March 2021, higher education was still teaching in this mode.

A large number of solutions have been implemented to limit the pandemic’s spread and try to return society and economic activities to previous “normal’. For example, the healthcare system moved to teleconsultation-based services using Internet of Medical Things (IoMT) tools such as digital medical remote consultation [19]. Another example, contact-tracing, and other means were deployed for identifying potential contact with a positively COVID-19 diagnosed individuals based on smartphone location, similar to Taiwan [20].

The Israeli population is massively taking the COVID-19 vaccine [21]. By 31 January 2021, more than 3 million people had received the first dose, and 1.73 million received the second one [22,23,24]. However, it is important to note in our context that young adults and middle-aged adults who are the most significant part of the high school and the higher education contingent started are less compliant to vaccinate [25].

This situation creates additional challenges for the educational system, higher education staff, and business managers. During quarantines and lockdowns [25,26], remote work may enable companies to maintain full or partial operational continuity if compatible with the required activities [27]. When on-site activities are inevitable, companies are legally required to restrict teams’ size and maintain persistent team members to control the spread of COVID-19 [12,13,14]. Comparably, remote learning of frontal courses can allow pedagogic continuity in education, but in the IEM students’ specific case, public health and social-distancing constraints have impacted their learning and training process. One of the most significant challenges encountered was finding ways to replace practice components, like laboratories and projects, with remote alternatives. These hands-on elements are essential for engineers’ training in general and industrial engineers, particularly since they foster innovation and develop students’ group interaction skills and managerial capabilities.

The case study presented in the paper aims at sharing with the Industrial Engineering and Management academics and professionals one Israeli experience of teaching and training the next generation of professionals in a context of complex and worldwide crisis.

Understanding the modernization of a part of the IEM curriculum in combination with its migration to a fully-based online landscape must help to understand how blended learning should be efficiently used when the engineering training involving, hardware manipulation, face to face group dynamics, and leadership skill development, will be back to normal without imposed social distancing.

The higher education system has been intensely engaged in the effort of emergency risk management and mitigation of the COVID-19 pandemic involving scientific research of delivering solutions from the health and business perspectives to overcome the crisis. One of the main contribution fields relates to the “on the move” innovation of the online education models [28]. One crucial aspect relies on redefining how to involve the academic staff in online teaching during and after the COVID-19 pandemic. One of the main results shows that for efficiently delivering online and distance learning content, the staff must show a higher interaction with students and adapt how to run assessments [29]. As we write these lines, the time horizon for the “return to normal” is very uncertain and a new set of skills must be developed to train students to manage technology development in a foreseeable future of virtual work environments.

Moving from face-to-face teaching/learning has induced a global reaction in the overall education systems. Over 31 countries have a joint reflection run during spring 2020 for sharing feedback on emergency remote education. The main focus was more on how social injustice, inequity, and the digital impacted learners and why the (higher) educational community must support each one for reducing the impact of being alone [30]. Additionally, one of the critical and recurrent points over countries and higher education systems relies on the lack of preparedness to deal daily with online teaching and implementing this kind of platform. One standard solution has consisted of asking non trained teaching staff to deliver online courses without being efficiently prepared for it. On the other hand, some institutions have canceled teaching activities for a few days to a few weeks to train their staff to move to distant learning by promoting experience sharing by the staff practicing online-based teaching in regular time [30]. The Israeli higher education providers have generally adopted this second alternative based on different projects implementing online teaching [31].

Industrial Engineering and Management departments worldwide moved their teaching both fully online for theoretical courses and in a blended way for the one involving practices [32,33] or moved both theoretical and practical courses online when possible. In some cases, they canceled the activities which cannot be provided remotely [34].

This study’s leading objective is to propose new ways to promote online and cloud-based technologies for teaching cyber-physical systems, both at the initial training and continuous one during the professional career. An additional objective is to recommend practical and real-world experimented ways to increase the quality and effectiveness of teaching and training engineering students to Industry 4.0 management and manufacturing technologies in regular and crisis times.

Three hypotheses lead the observational research (case study) presented in the following sections:

- The COVID-19 pandemic allows for accelerating industrial engineering and management changes, such as blended teaching, practice, and mentoring as a standard.
- Moving to successful simulation or online cyber-physical systems as a part of industrial engineering and management education and training depends on reactivity and adaptability of the teaching staff and the students.
- Industry 4.0 deals with distant actuation monitoring and management. Therefore, Online-based learning that covers all aspects of the domain is fundamental to train the next generation of engineers and managers and provide the needed skills.

This manuscript focuses on relating the real-world experience of upgrading the IEM curriculum before and during the COVID-19 pandemic. The goal is to point out the importance of involving the students in their training in a tangible way. This involvement is the critical way the studies occur in a dematerialized context, but similarly to the new reality of remote working and multi-site exploitation. This research characterizes changes needed in teaching and learning Industry 4.0 theories, concepts, and systems to facilitate graduates’ integration in the employment market.

Section 2 presents the methodology used to upgrade the IEM curriculum and take it forward to Industry 4.0 before and during the COVID-19 pandemic. Section 3 presents different updated and upgraded courses and capstone projects of the bachelor program focusing on Industry 4.0 and smart manufacturing. Section 4 discusses successively (a) how the education and research infrastructures have been adapted for supporting distant-learning, practice, and research, (b) the strengths and limitations of the IEM upgrade disclosed in the present manuscript, (c) current and potential future directions. Section 5 summarizes the main conclusions of this observational research.

## 2. IEM Curriculum Update Methodology

As a modern discipline, Industrial Engineering and Management (IEM) is at the crossroad of many fields such as engineering and technology, formal and natural science and management science, and human sciences.

Management, as a transverse component of IEM, is organized over five central concepts [31], which are:

- Forecasting, looking for innovation, future potential of market development, imagining scenarios that may occur, and defining actions for handling new challenges.
- Planning, defining, deciding, and scheduling actions to deal with the previously forecast challenges and bring them to goals and action plans.
- Organizing by allocating human and non-human resources for acting according to the plan.
- Coordinating, achieving the different steps of the action plan and goals.
- Controlling, checking that research, development, and production activities fit the disruptive goals and progress accordingly to the strategy previously defined.

Industrial engineering is commonly defined as a domain attending to developing and improving different kinds of business and industrial tasks, e.g., describing a modus operando; and handling milestones of a production. Offering an up-to-date and cutting-edge curriculum is challenging. It is required to consider the curriculum of other engineering specialties to give the students the ability to communicate and be productive with other specialists efficiently. Furthermore, as IEM is a very dynamic field and with the continuous development of new technologies, the students’ training must fit industry needs both locally and globally. Thus, the methodology for developing, evaluating, and maintaining a set of syllabi dealing with Industry 4.0 and smart manufacturing must consider the following in regular times:

- Evaluating the syllabus, in regard to the current and predicted evolution of the industrial and business environments;
- Proposing improvements taking into account both, the expected changes in the industrial and business environments, and the academic budget and infrastructure constraints;
- Getting previous years students and graduates’ feedbacks, and understanding their needs in regards of their involvements in none-academic projects and positions.
- Getting peers and industrial practitioners’ feedback, by submitting the syllabus proposal to faculty and institutional committees involving these people giving suggestions for consolidating the new syllabus;
- Implementing the new syllabus during the next relevant academic semester;
- Getting feedback and suggestions during course run for the students and eventually teaching assistants;

Taking into account the above and dynamically updating the course content in the COVID-19 crisis context and the forced move to full-online-based teaching, practice, and mentoring, this framework has been adapted at the faculty level in the shortest downtime of one week. The methodology we used for adapting, enhancing, and evaluating the learning and teaching experience under the urgent and unexpected challenging request to move from face-to-face university courses to be taught distantly and online [25] is as follows:

- Considering the recently upgrade courses before COVID-19 implemented for the first time during the fall semester 2019–2020;
- Moving to emergency-mode management and upgrading relevant syllabi for delivering the most similar course content during COVID-19 lockdowns;
- Semestrial students’ feedbacks (before and during COVID19 pandemic);
  ○ Before the COVID-19 pandemic, the student had to complete at the end of the course a survey;
  ○ During COVID-19 lockdowns, to reduce the risk of student’s churn, the faculty’ management frequently interviewed students for getting their feedbacks in near-real-time;
- Learning from experience and moving forward by preparing the post-COVID-19 teaching/learning era (will be considered in the discussion section below in a transversal manner).

It is important to highlight that focusing on the COVID-19 pandemic higher education crisis, an equivalent case study focusing on a set of syllabi dealing with Industry 4.0 and smart manufacturing has, to our current knowledge, not been published.

## 3. Results

Before the pandemic and the needed social distancing rules (associated with national confinements), the overall curriculum was taught in a frontal manner.

The curricula were initially drafted and approved before the beginning of the pandemic. Accordingly, these “contracts” between the teaching staff and the students cannot be deeply revised at short notice. If not carefully designed and monitored, they may induce unexpected changes in the achievement of educational objectives and goals. Distant, online, synchronous/asynchronous teaching and learning is a new standard in the COVID-19 era.

The following section describes the courses and the specific adaptations during the last year (since March 2020) to respond to the urgent COVID-19 demands. Given the present situation worldwide at March 2021, it is reasonable to forecast that these changes will have a mid-and long-term impact on teaching IEM as a part of the business and industrial ecosystem-induced changes that now encourage remote working when possible [35,36].

This section focuses on four courses in the IEM curriculum at HIT and the graduation project that are at the heart of the educational requirements of the fourth Industrial Revolution:

- Introduction to algorithmic and Python (Python);
- Computer Integrated Manufacturing (CIM);
- Introduction to Internet of Things (IoT);
- Models of Business Intelligence (MBI);

These four courses and the graduation capstone project (GP) are linked to one another and are a part of the third and fourth years of the curriculum. Their related project-based learning approach emphasizes the viewpoints of both standards, “Reference Architecture Model Industry 4.0—RAMI 4.0” and “Industrial Internet Reference Architecture—IIRA” [37,38,39]. RAMI 4.0 and IIRA are essential references and key architectures of smart manufacturing, a central pillar of the Industry 4.0 era. Both have a similar goal: providing a framework for digitizing industrial operations and optimizing the manufacturing processes through technology. RAMI 4.0 and IIRA diverge in their application scopes. RAMI 4.0 mainly relates to the manufacturing value chain, while IIRA particularly refers to building, implementing, and operating IIoT systems in any industrial field (e.g., energy, healthcare, manufacturing, transportation). Each one, in its way, deals globally with system functionalities and architecture, its implementation, and its monetization (from the RIMA4.0. perspective “life cycle & value stream”, and from the IIRA one “business”). Project-based learning as a part of each of the listed courses above allows the student to enhance and apply his/her knowledge research-oriented and real-world tasks. RAMI 4.0 and IIRA are standards used in the industry, and the students are encouraged to develop and implement their projects by using them. Let us now look at each course and the graduation project in some detail.

### 3.1. Introduction to Programming in Python

#### 3.1.1. Before COVID-19

Introduction to Programming in Python is an introductory course for undergraduate students in the Industrial Engineering and Management program. The course introduces basic principles of modern programming techniques and problem-solving logic. The course deals with the practical aspects of programming and imparts Python programming skills. The course includes an introduction to the concept of an algorithm, basic variable types, operators, courses, conditions, loops, regular and recursive functions, methods for passing parameters, data structures (one-dimensional and multidimensional lists, tuples, and dictionaries), and interacting with text files. The programming language we use in this course is Python, which is a popular programming language. Python is a dynamic, object-oriented language and has an elegant and friendly syntax for the novice programmer. Before the COVID19 era, the course had been delivered in four academic hours: two hours of theory (using slides and whiteboard) and two hours of practice in a dedicated laboratory. During the practice course, the students solved given exercises and then compared them to the slides’ solutions. During the independent work, the students asked questions and asked for direction.

#### 3.1.2. During the Pandemic

Moving to emergency-mode management at the beginning of the pandemic, the course is taught using video conferencing software. The theoretical part of the course required only minor adaptation by adding further explanation slides to the presentation.

The main challenge was the practice part of the lessons, which required significant changes. First, more solved examples were provided and explained in the plenary session. Next, during self-practice, the students got exercises, and they were allocated to breakout rooms, 3–4 students in each room. The lecturer moved between virtual spaces, where the students could share their screen, and provided assistance and direction. Besides, the students in the room discussed the exercise and helped each other.

#### 3.1.3. Students’ Feedbacks

The feedback was very positive about the theoretical part of the online course. The students claimed that the remote mode is more efficient, in the sense that more material was covered and more examples given. However, the students lamented about the practical part of the course. They commented that in remote mode there is not enough personal attention and help with code bugs, that is possible in frontal lessons.

Regarding the lack of practice, Moodle offers some modules, such as “VPL—Virtual Programming Lab” [40] is an activity module providing programming assignment management. It will be implemented, in this course, as a way to track the progress of the students in their learning journey most significantly and as a way to provide each one the relevant task supporting her/his progression.

### 3.2. Computer-Integrated Manufacturing

#### 3.2.1. Before COVID19

The CIM course gives the students the fundamental tools for designing, implementing, and managing automated production environments and their computerized control systems. CIM is a semester course (13 weeks) of three lecture hours a week and one laboratory hour a week, combined with Project-Based Learning involving teams of two to three students. This course is articulated around three main components:

At a first step, the students are getting an overview of industrial process and control, in parallel with introducing the history of the different industrial revolutions and their impacts on day-to-day life. This part is associated with refreshers on essential mathematical tools needed to deal with automation and introductory physics to understand how sensors operate. The second principal component of the CIM course focuses on teaching “how-to”:

- Draft and implement specification of discrete and continuous control applications;
- Use information technologies for processing industrial data and supporting decision making, for example, for handling troubleshooting of production lines;
- Use Supervisory Control and Data Acquisition (SCADA) systems for integrating the control of discrete and continuous (sub-)systems;
- Smartly use at the production plant and end-business mobile asset tracking technologies (e.g., bar-codes, Quick Response (QR) codes, near field communication (NFC), and radio frequency identification (RFID).

These two first parts of the course were before the COVID-19 pandemic events, teachable in a non-frontal way, and mainly involving the students by using different simulation software environments. In the third part of this course, the students discover “robotics” as an automation kind and how it can be integrated into the production processes at small and large scales. The first lessons are herein mainly dealing with the history of robots, ethics, the safety of use, and physics concepts such as direct and indirect kinematics, motion limitations, and the integration with machine vision tools.

**Figure 1:**
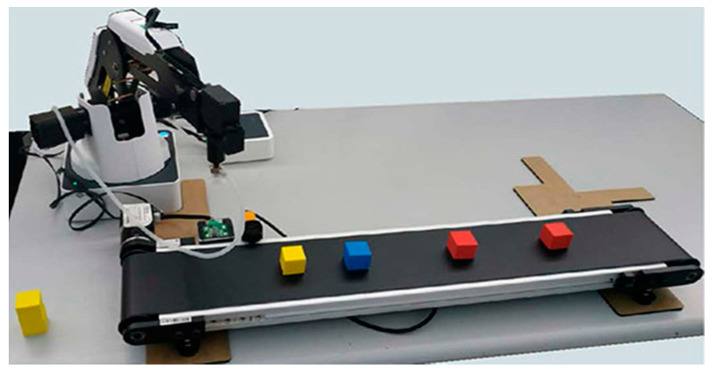
Dobot Magician and its rail in a robotic lab course.

All these concepts are more easily gained when the students are participating in lab workshops. Thus, they are actively involved by using an arm robot such as Dobot Magician (Figure 1) and its educational software (i.e., a suited visual programming version of Blockly [41]. The main objective is to ensure fast adoption of the robot tool, and so experimenting its behaviors and constraints). In regular times, the overall course takes place in the AURIS lab (see Section 4), allowing working both on software and hardware in the same place with high computational resources.

#### 3.2.2. During the Pandemic

To avoid meetings and respect social distancing, the robotics workshop used an education robot simulator (i.e., CoppeliaSim Edu [42]). Moreover, many students have designed and built simulations of cyber-physical systems, following the IEC 61131-3 standard [43], focusing on solutions supporting industrial and general public customers during the pandemic. During the pandemic, the development of simulated systems by the students introduced them the concept of “digital twins” [44], which allows, e.g., designing of the configuration, motion, control, and optimization model of a flow-type smart manufacturing system [45] and then a possibly rapid reconfiguration of an automated manufacturing system via an open architecture model [46].

#### 3.2.3. Students’ Feedbacks

Before the COVID-19 crisis, the students’ feedback mainly pointed out the need to concentrate the laboratory and practice time on larger slots (2 h every two weeks instead of one hour per week). The largest part of the students was highly and intensely engaged in this course’s PBL experience. It allowed them to prepare themselves (according to their feedback) for the graduation project and industry (when they were regular track students).

This course has successfully moved to an online version, getting positive feedback from students with and without a prior background in automatics and electronics (e.g., vocational graduates). However, practice with hardware has this course’s PBL experience been reported as a missing part of the course; even the use of simulation software was strongly expected and supported by the teaching staff.

### 3.3. Introduction to Internet of Things

#### 3.3.1. Before COVID19

The course is optional and is the continuation of the CIM course, which is mandatory.

It allows students to study in-depth some critical components of the fourth industrial revolution, the IoT. IoT brings together smart objects, cloud computing, and automated analysis of large amounts of data. This introduction to IoT mainly deals with the hardware and cloud computing aspects [9].

The course presents the history of IoT, the status of the field today, key initiatives and key players in IoT, and teaches about the significance of the implementation of smart and interactive devices.

The main subjects of the course are:

- An introduction to IoT [47] and its history, from machine-to-machine (M2M) system solutions to IoT solutions [48,49,50];
- An overview of key enabling technologies [51];
- Service-Oriented Architecture for the design of IoT systems [52];
- Applications in the industry [53,54,55].
- A more in-depth analysis than in the CIM course of the mode to operate of the different sensors, cameras, and actuators;
- Prototyping IoT systems [56].

A large part of this introduction to IoT is dedicated to practicing. Therefore, students are working with Raspberry PI kits which make the hardware more accessible to the students. Raspberry PI is a friendly single-board computer that students can quickly turn into IoT product prototypes [57]. Moreover, during this course, the students improve their programming skills and knowledge of the Python programming language [58].

#### 3.3.2. During the Pandemic

Since the COVID-19 crisis, the course was delivered online by using a video conferencing platform. The new reality impacted the program by introducing two main and strong constraints, the students:

(1) could not use the dedicated IoT laboratory
(2) could not take home the IoT equipment for practice. Therefore, significant adaptations have been performed to the course curriculum, increasing the focus on:
  - Systems modeling: Unified modeling language (UML) was added to the curriculum [59] and was taught deeper than in mandatory courses. These extra lessons enabled the students to delve deeper into the design of IoT solutions
  - Simulation: since the students were not enabled to build an IoT product using dedicated software, they learned how to simulate an IoT product using Python programming language and particularly by using the “Faker” package that generates random users and random data with the programmer (i.e., the student) defined constraints [60].
  - Cloud services: besides essential AmazonWeb Services (AWS) as S3, Simple Notifications Service (SNS) and Simple Email Service (SES), additional services have been introduced as AWS Lambda and AWS Rekognition.
  - Projects with emphasis on COVID-19: during the course, the students must design, use UML and SOA models, and develop an IoT product using cloud services and python. Students were instructed to design and develop a project related to COVID19 or lockdown-associated issues.

#### 3.3.3. Students’ Feedbacks

The course was offered during the winter semester; the third one taught during the pandemic. Therefore, the students already have had experience with online courses.

The fourth and senior students of this course expressed their preference for the online mode. They were delighted with theoretical lessons, and practical experiments run remotely using their computational resources. We received feedback such as “The course was exciting and delivering innovative content” and “Despite the online and challenging learning, the course trained us to new technologies”.

However, the student lacked real-hardware practice. They mainly had previous experience working according to the PBL methodology, in the third-year course dealing with CIM, and engaging them in regular time (pre-COVID-19 era) with hardware (e.g., small robots).

### 3.4. Models of Business Intelligence (MBI)

#### 3.4.1. Before COVID-19

**Figure 2:**
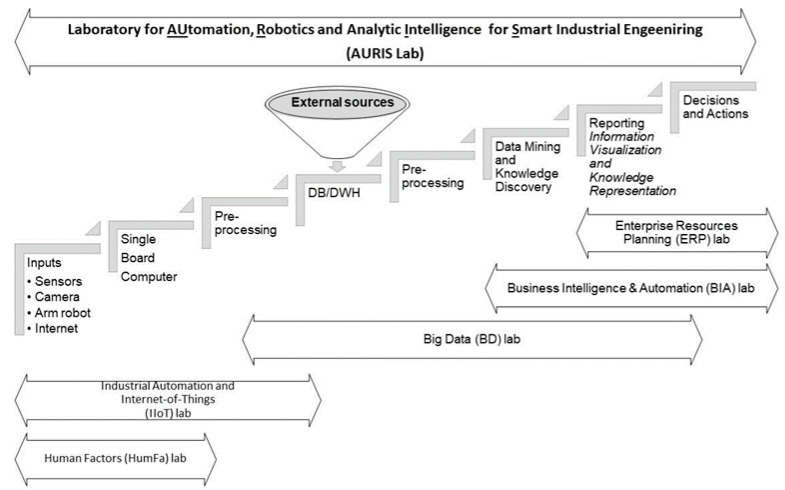
The flow of the HIT Technology Management lab cluster.

Business intelligence [61] supports data-driven decision-making by integrating the knowledge discovery process’s overall steps [62,63]. Figure 2 shows the knowledge flow from data collection to actions from an industrial engineering and management perspective. Accordingly, the “Models of Business Intelligence” course is a fourth-year elective course. The course provides students with basic knowledge of how business management systems and engineering models and business intelligence work. It presents the operation principles of business intelligence systems, emphasizing these systems’ methods and tools. Students examine a series of business problems during the course and solve them with BI tools such as Microsoft Power BI [64,65]. More specifically, this course is project-based learning-centered. Therefore, the students must develop a business intelligence solution in the context of a business or industrial collaboration or their graduation project as a reporting and decision-support component.

#### 3.4.2. During the Pandemic

During spring 2020, this course has been run entirely in an online version in webinars format and recorded tutorials. The flexibility of Microsoft Power BI as a free tool and a low-cost online platform allowed students to work on their project efficiently and professionally, giving senior students just before graduation an advantage on their resume, particularly in troubled times COVID-19 pandemic.

#### 3.4.3. Students’ Feedbacks

In the previous years (in the pre-COVID-19 era), the students’ feedback was very positive, and the engagement and involvement in the PBL were significant. In their last semester of B.Sc. studies, the students received inputs regarding the course utility in their position search in the industry and more about the recruiters’ interests during the course and its integration in the graduation one. Indeed, the PBL activity of the NBI course is, in most cases, the last part of the graduation project, and the students are using both as a part of their project’s portfolio.

The “Models of Business Intelligence” course is one of the first courses that moved online in the shortest delays regarding prior experience in delivering a part of it online in synchronous and asynchronous ways. From the students’ perspective, the online version of the course got positive feedback. Moreover, to help the students benefit efficiently from completing the PBL activity, the extra-hours dedicated to supporting their training to Power BI have been increased with specific time and assistance to each project.

### 3.5. Project-Based Learning and Graduation Projects

All the courses discussed in this manuscript give a strategic and central position to PBL. PBL is a fill-component of the new pedagogical approach of the upgraded curriculum. Moreover, students are developing their skills over different courses by starting their PBL journey in one course, for example, CIM, and go deeper during another one, such as the IoT, and complete the same project as a part of the final graduation project requirements.

Before the COVID-19 pandemic, social distancing, and the different confinement waves, the faculty lab cluster for teaching Industry 4.0 specialties (i.e., Automation, Robotics, IoT, Big Data and Knowledge Management, Human-Machine Interactions, Artificial Intelligence, Business Intelligence, and Analytics) ran flexibly. The regular, evening, and visiting (from aboard) students could schedule with a research assistant to develop and implement their PBL and graduation projects.

- AURIS—Automation, Robotics, IoT, and Analytic Intelligence for Smart Industrial Engineering,
- IIoT—Industrial Automation and IoT
- Big Data—Big Data,
- BIA—Business Intelligence and Automation,
- ERP—Enterprise Resources Planning,
- HumFa—Human Factors.

As it is presented in Figure 2, the lab cluster deals with:

(1) Data acquisition from different kinds of inputs, such as sensors and their related small board computer, robots, social networks, and more,
(2) Data cleansing,
(3) (Big) Data Management,
(4) Knowledge Discovery with Data Mining, Text Mining, Process Mining, Machine Learning, Statistics, Natural Language Processing, to
(5) Knowledge Management, and
(6) Decision Support making.

The cluster activities relate to industrial and business applications such as automation, industrial manufacturing [66], services, healthcare [67], safety, and quality.

The AURIS laboratory is the most recent addition to the cluster. It integrates the Industrial Engineering and Management faculty curriculum’s overall educational activities with a strong focus on Industry and Automation 4.0. The robotics lessons in the CIM and IoT courses are involving the use of low cost educational and production systems combining, for teaching the end-to-end computational and Information Management processes:

- Small robots (e.g., DoBot Magician) [68],
- Single Board Computer assemblies (e.g., Raspberry PI) and their related sensors and actuators [69],
- Cloud technologies (e.g., Amazon Web Services, and Microsoft Azure—Cloud Computing Services [70],
- Knowledge Management and Business Intelligence platforms (e.g., Microsoft Power BI) [71].

Accordingly, the IIoT laboratory focuses on sensors, controllers, actuators, a device-to-device connection, wireless technologies, mesh communication networks, and low-cost computerized manufacturing systems (such as small robots) [10], as well as quality and safety in the industry. The IIoT laboratory teams teach courses such as Introduction to IEM, Algorithmic and Programming, Automated and computerized manufacturing, Internet of Things, and sensors.

The BIA laboratory research emphasis on artificial intelligence and business intelligence applications, mainly from the data science perspective, with a focus on data mining, machine learning, language processing, knowledge engineering, management, and representation (terminologies, taxonomies, ontologies, information visualization), data quality and quality in information systems. The BIA laboratory researchers are involved in teaching activities related to research such as analysis and design of databases, data mining, machine learning, big data and cloud technologies, business intelligence models, quality engineering for information technology, and digital health.

The creation of the lab cluster was raised by significant developments in the faculty activities such as:

- Students’ increasing interest in carrying out their final graduation projects in an academic framework and applied technological research in the domains investigated by each lab alone or overlapping. Some of these projects have a strong potential for becoming industrial developments.
- International cooperation in research, teaching, and student mentoring.
- Enhancement of research, teaching, and mentoring actions intersecting between the cluster and partners’ labs inside and outside HIT.

Moreover, this lab cluster and its continuous enhancement for considering the recent advances and the new perspectives in the Industrial Engineering domain aim to deliver an end-to-end vision and understanding of the Organizational Information systems and Knowledge Management resources [71]. Accordingly, it supports the development and implementation of academic staff researches and student graduation projects. Below, examples of undergraduate students’ projects following this model are given.

#### 3.5.1. Project-Based Learning before COVID-19 Crisis

Upgrading the IEM curriculum specifically from the automated systems perspectives (CIM, IoT, and BI) was associated with an increased central position given to the project- and problem-based learning approaches. Below two examples of students’ accomplishments in this framework, as graduation projects:

- Smart household pet feeding monitoring system—A design and implementation of a pet feeding station prototype. Additionally, it provides health and behavior monitoring services, gathering health’ pet indicators such as feeding habits and weight [10].
- Smart parking system design—Design a network of cameras and motion sensors that collect data about parking lots throughout the city. All devices are powered by solar energy and are connected wirelessly to a local Raspberry Pi. The Raspberry PI uses cellular communication and transmits the data to Amazon servers. The system’s purposes are on first-hand billing drivers for legal parking around the city and to send out fines for illegal parking; on the second-hand monitoring available parking spots for the benefit of drivers.
- Smart baby monitor—A design and implementation of a monitor embedded with a microphone and camera connected to a Raspberry Pi. The monitor detects noise in the room and sends a short video of the baby to parents through Amazon SNS (Simple Notification Service).
- Smart garbage can collector—A system design of weight sensors that are attached to garbage cans. The sensors weigh the can and, using a connected Raspberry PI and cellular connectivity, report the remaining garbage can capacity (or lack thereof) to the local municipality.

#### 3.5.2. Projects Started during the COVID-19 Pandemic

As previously highlighted, the B.Sc. The COVID-19 pandemic has deeply impacted IEM studies. All the teaching activities moved online, such as practicing and developing engineering and managerial capabilities. Accordingly, by taking into account that the future graduates will have to start their career in the challenging COVID-19 era or post-era, the PBL framework has been adapted for:

- having a training continuity,
- reduce the least possible disruption of the educational activity,
- preparing the students to massively remotely work and dealing with non-materialized resources such as models and simulations.

Following, two examples of third and fourth-year (courses and graduation) projects specifically handling issues related to the COVID-19 pandemic.

COVID-19 vaccine containers: the project consisted of following the dispatching of COVID-19 vaccine doses. It has been defined as a complex logistic end-to-end process. Indeed, these vaccine doses need to be stored at a low temperature continuously, and they are exposed to a hijacking risk. Similarly, to the solution proposed to the vaccine producers, some of our students have designed and simulated computationally and (no physically implemented because of national confinement and restriction of access to the campus) a container with sensors and actuators connected to communication systems and providing:

- monitoring of temperature, light intensity, humidity, vibrations, falls, and openings of the said unexpected container;
- position tracking services, alert generation, and push in case of unexpected deviation of a predefined route;
- user identification (i.e., accredited individuals to receive and to open the container);
- real-time reporting of the status, state, and position of the container.

Transportation and hygiene: This project intended to design an online application for hygiene reports in the public domain, like malls and public transportation, and private venues such as restaurants. During COVID-19 times, when maintaining hygiene and social distancing is very important, technological and collaborative tools can contribute significantly to this common goal. The relevant existing technological platforms are not developed enough [72]. This application helps maintain public health by providing up-to-date information on the cleanliness of the physical space. The application will be used by both the general public and the venue owners.

This project involved acquired technical knowledge and skills during the different courses disclosed in the present manuscript. This kind of PBL allowed the students to understand this kind of product’s potential genericity, which may have other applications such as transplantation organs or sensitive samples transportation.

### 3.6. Students’ Satisfaction Survey

The students’ satisfaction with the teaching process is measured in regular time at the end of each semester for each course. The questions of this survey are the same, each semester, for all the courses taught in our institution. The survey focuses on:

- the organization and clarity of the teaching material;
- the way for delivering the course in an interesting manner;
- the way to involve and induce active participation of the students;
- the quality of the communication between the teaching staff of the course and the students;
- the feelings about the contribution to the learning experience of the home-work or course projects;
- the overall satisfaction about the course.

During 2019–2020, only at the end of the fall semester (February 2020) a computed and anonymized survey has been run. During the spring and summer semesters, instead of running students’ satisfaction surveys, in order to increase the numbers of contacts between the teaching and management staff with the students, the faculty’ staff frequently interviewed the students’ representatives for getting their feedbacks in near-real-time about each courses and the way to facilitate their learning experience. The feedbacks of these interviews are reported above for each course.

**Figure 3:**
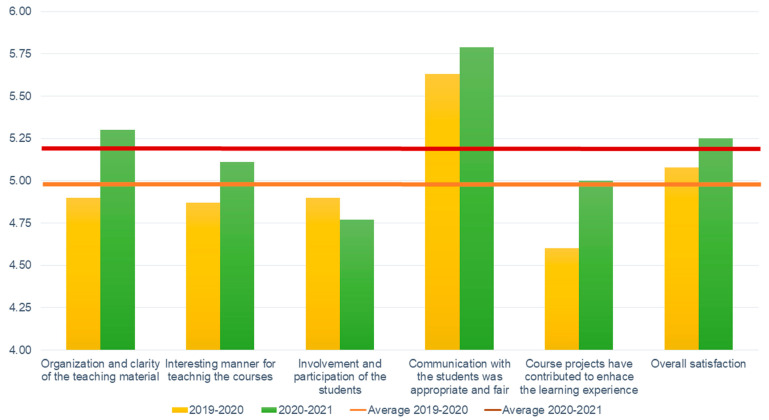
Synthesis of the students’ satisfaction surveys for the Computer Integrated Manufacturing and Internet of Things courses.

At the end of the fall semester of the 2020–2021 academic year, a computed students’ satisfaction survey ran. This survey is relevant for the “Computer-Integrated Manufacturing” (around 70 students each year) and the “Introduction to the Internet-of-Things” (around 15 students each year) courses taught during this period. Figure 3 shows the averages scores to the questions listed above for these two courses before and during the COVID-19 pandemic.

These results shows surprisingly and globally a satisfaction increase regarding these courses. Indeed, even the courses were delivered online and the course material organization in the learning management system has not change year-over-year, the students satisfaction increased (before vs. during COVID-19 pandemic: +8.16%). Similarly, the students which learned online have feel the courses contents more interesting that the one which learned it in-class (+4.93%). The same trend appears regarding the perception of the “project-based learning” approach (+8.70%). More, even than the courses were taught online, the students reported feeling a more appropriate and fair communication that the in-class students (+2.84%). However, we can notice a small decrease in the involvement and active participation feeling (−2.65%). The overall satisfaction has significantly increased (*p* < 0.05). It is critical to highlight, that even “Computer-Integrated Manufacturing” and the “Introduction to the Internet-of-Things” are engineering courses which are in regular time hardware-based the students report their overall high satisfaction. From an engineering perspective training the next industrial engineers and managers to Industry 4.0 during the COVID-19 era and so mostly online in our case is both challenging. Nevertheless, it is opening opportunities to look at the future of the whole Industry 4.0 and the online, distant and cloud-based operations [73].

## 4. Discussion

Training the next industrial engineers and managers about Industry 4.0 in regular times is a challenging task. The COVID-19 pandemic added specific additional challenges and opened new opportunities for accelerating the curriculum and supporting infrastructure transformations.

### 4.1. Faculty Lab Cluster for Teaching Industry 4.0

The COVID-19 pandemic has crucially impacted the educational and the research activities of the lab cluster, which is built around six laboratories. During the first months of the COVID-19 pandemic to provide to the academic staff and the students continuity of services, a large part of the activity moved online like teaching activities. More specifically, the overall computational activities moved to online services: Microsoft Power BI for Business Intelligence, Amazon Web Services for Artificial Intelligence and IoT activities, to offline simulation systems using CoppeliaSim for Robotics, and to remote access to the HIT Big Data management systems. However, the hands-on practice learning with hardware equipment in the IIoT and AURIS labs has been sensibly impacted and used only in singular graduation project cases. Section 4.3 below presents our present efforts to overcome these difficulties.

### 4.2. Strengths and Limitations

COVID-19 pandemic and crisis are playing the role of a kind of revolution starter and innovation booster in the academic [74], public service delivery [75], and business [76,77] fields.

Industrial engineering and management is a modern science at the crossroad of numerous fields. The Technology Management faculty’s multidisciplinary focus at HIT provides the students with the knowledge and skills to be involved in complex product development in continually changing environments. At their future position, they need active collaboration with electronics, automation, and robotics engineers.

Recently graduated students, which were the first ones experimenting in 2018 with the study program’s changes, share their helpful feedback. The experience they gained in their final projects related to CIM and IoT contributes to their real-life working environment (e.g., quick real-world project understanding and involvement, rapidly getting managerial responsibilities).

Although we recognize that it is by no means an indicator that can be shown as statistically significant we supplement the quantitative feedback presented in Section 3.6 by sharing some original students’ feedback statements without any editing, save the italics for completeness, to illustrate their feeling:

(1) Examples before the pandemic:
  - Classroom exercises contribute to understanding.
  - Add a short explanation in presentations in a situation of someone who did not come to class.
  - Many examples were presented throughout the course.
  - Presentation of a topic by students (*are useful*).
(2) Examples during the pandemic:
  - More practical examples, more practice (*required*).
  - Remote teaching and the possibility of repeating the lesson when you want to deepen (*knowledge*) while answering homework give amazing tools for study even outside of school hours.
  - An online course is better because there are a lot of class discussions and division into groups—something that cannot be held in the classroom.
  - Continue zooming even after the “COVID-19”.

One of the main challenges observed is students’ heterogeneity in background and interests. Most of them do not have knowledge and experience in electrical or mechanical engineering. It is the first time they work with and assemble hardware and control robots. The updated program tries to make the hardware available to students by introducing them “user” friendly components.

Moreover, we are working on integrating the PBL approach into the overall curriculum. At this time, as pointed out above, the “Introduction to programming”, CIM, “Introduction to IoT”, and “Models of Business Intelligence” courses afford the students work on a joint courses project, allowing them to apply their new knowledge and skills progressively. The program will offer them a transverse and joint project to several technologically linked courses to answer market requirements for graduates with multidisciplinary and integrative expertise. The lab cluster will allow interdisciplinary learning and research [78]. Also, we are currently developing multidisciplinary courses and seminars with other HIT faculties, integrating IoT, Robotics, Educational Technologies, and Digital Health [79], or more widely, Industry 4.0.

### 4.3. Current and Potential Future Directions: Lab Cluster Virtualization and Digital Twins

An essential part of upgrading the industrial engineering and management curriculum for the Industry 4.0 reality is increasing international collaboration projects and student exchanges at the faculty level. Therefore, the faculty and its lab cluster are hosting physically on regular days and remotely during the pandemic times, students and research staff from leading higher education institutions from each one of the five continents.

Additionally, in the COVID-19 context, social distancing is a norm, and sanitizing the workspace frequently is required. This need has accelerated the development and implementation of an innovative and advanced system supporting operating the laboratory infrastructure (robots and similar IoT systems) remotely. This must allow students and staff to interact efficiently to acquire practical skills equivalently to frontal lab work. Research related to the virtualization of laboratory resources for encouraging and facilitating the study of technological processes has been described in the literature, in different modes, for the last two decades [80,81,82,83].

In the context of the upgrading actions of the industrial engineering and management curriculum for fitting with the needs of Industry 4.0 and the post-COVID-19 era, the authors have set up a challenging postgraduate project at the beginning of 2021 to produce a working prototype of a virtual lab that can be implemented from the home computer of staff and students. The project is underway as we write this manuscript. In brief, the setup will be operated from a home computer. It will include a connection via the network to a remote gateway at HIT to which microprocessor endpoints will be connected wirelessly to sensors collecting data such as temperature, humidity, noise/sound, and images and, not less critical, robotic actuators that can simulate the human operations in the lab. The technological solution will work through IoT-compliant communication protocols and add-on hardware to the existing equipment that allows the entire network to work in a close-loop without human presence in the lab. This is aligned with the higher education regulation in Israel that emphasizes a digital ecosystem transformation to a system expected to adapt to a continuously increasing number of students and limited resources such as infrastructure and staff. Moreover, the project is performed in a framework of international cooperation with universities in Europe and Argentina that have identified similar urgent needs. It is expected that a prototype will be working for the coming 2021 summer semester to enable the first pilot run of the IIoT course in a blended virtual and physical mode. Upgrading the industrial engineering and management curriculum for Industry 4.0 and handling the numerous challenges of the COVID-19 era pushed the boundaries forward at the next level. The education of the 21st century Industry 4.0 engineers has to perform in a continually changing environment.

As a next step, and for enhancing the usages rate of the lab cluster infrastructures, we are investigating existing solutions for sharing our local resources and getting access to devices and resources not available on our side. For example, platforms such as LabsLand [84], WebLab-Deusto [85], or DigiLab4U [86] allow connecting educational institutions with their own real laboratories or one of the platform users somewhere else around the world over the Internet. Moreover, augmented and virtual reality can give over this kind of platform the possibility to give the filling of controlling a device on-site, such as a robot. The added value of this kind of platform [84,85,86] is that their users (students and researchers) are not working with a simulation platform but a real cyber-physical system. In some way, using this product of our research and the existing platforms discussed above leads to use real implementation of “digital twins” [44] and preparing the students to engineer and to manage complex systems in an abstracted and virtualize way like advanced systems work nowadays, for example, in agriculture [87] or healthcare [88].

## 5. Conclusions

The case-study reported in this manuscript addresses the flow from system design to implementation areas of an industrial engineering and management curriculum, including practical examples, feedback received, and the rapid decisions and changes that have been made to encompass the effects of the COVID-19 pandemic on content and teaching methods.

Three hypotheses drove this observational research:

Firstly, we demonstrated that the COVID-19 pandemic (more than any other significant events) allowed accelerating changes to educate and train the next generation of industrial engineers and management. As a concrete example, the proportion of staff teaching online (at least in a blended approach) jumped, at the faculty of Industrial Engineering and Technology Management at the Holon Institute of Technology, Israel, from 10% before the COVID-19 pandemic to 95% during the first-week s of the crisis then 100% today. Moreover, when teaching returns to normal (with reduced social distancing), all the students will have to learn at least two online courses during their curriculum. Furthermore, and this the most critical point herein, the students were delighted with the online framework, allowing them access to recorded lessons and giving them a higher time management flexibility.

Secondly, we highlighted that moving efficiently to simulation or online cyber-physical systems as a part of the industrial engineering and management education and training to smart manufacturing and Industry 4.0 depends on reactivity and adaptability of the teaching staff and the students. Therefore, the students were significantly (*p* < 0.05) more satisfied by online teaching than in-class. This must be due to the flexibility of the teaching/learning process that can fit with familial and professional constraint during quarantine or confinement times. It is also possible to envisage that the virtual lab we have started to implement may also improve the indicator of “involvement and participation” that was the only one with negative tendence in results in Section 3. Nevertheless, we also noticed that this transition must be a part of a “disaster” preparedness plan or, in other words, needs to be a whole scheduled and organized process of the evolution of the IEM curriculum and the infrastructure upgrade actions.

Thirdly, online-based learning in the industrial engineering domain is critical to train the next generation of engineers and managers. Accordingly, the development and the use of platforms allowing working with real-distant systems prepare the students to Industry 4.0. They will be able effectively and efficiently take their future responsibilities “on-filed” by having a real-prior experience with remote working and management (e.g., via SCADA systems, cloud-based computing, robot control). Besides, the use of blockchain technology must provide a layer that enables enhancing traceability of actions over these platforms and systems, increasing the security of access [89], and so allowing reducing the utilization costs by facilitating the optimization of resources management [90,91,92]. This blockchain-accountable approach must empower sustainable manufacturing, product lifecycle management [93], and intellectual and industrial property by reinforcing anti-counterfeiting control processes [94].

The results of the use-case presented in this manuscript can be generalized to any industrial engineering related domain, such as electrical and electrical engineering, systems engineering, software engineering, and more by considering that online teaching based on simulation and remotely shared resources allows students to by efficiently trained to the new business and industry features that will emerge in the post-COVID-19 era. Moreover, the curriculum update process before and during the COVID-19 pandemic allowed to point-out the crucial necessity to develop “disaster preparedness plan” curriculum fitting with the (industrial 4.0 and manufacturing) engineering needs at the present and expected future Industry needs

Finally, training the next industrial engineers and managers to Industry 4.0 is challenging over the COVID-19 triggered chances. Nowadays and in the next few years, Industry 4.0 will be led by the artificial intelligence, blockchain, cloud/cybersecurity, and data analytics (ABCD) paradigm. These four technologies converge to a multidisciplinary focal-point orchestrating innovation in many businesses and industries [73]. The lab cluster supporting the industrial engineering and management curriculum upgrade at the Holon Institute of Technology considers the ABCD paradigm by addressing each of these disciplines in at least one of its laboratories.

## Data Availability

The data presented in this study are available on request from the corresponding author.
